# μ-1,1′-Bis(diphenyl­phosphino)ferrocene-κ^2^ *P*:*P*′-bis­{[(*Z*)-*O*-isopropyl *N*-(4-nitro­phen­yl)thio­carbamato-κ*S*]gold(I)} chloro­form disolvate

**DOI:** 10.1107/S1600536809043864

**Published:** 2009-10-31

**Authors:** Soo Yei Ho, Edward R. T. Tiekink

**Affiliations:** a Department of Chemistry, National University of Singapore, Singapore 117543; b Department of Chemistry, University of Malaya, 50603 Kuala Lumpur, Malaysia

## Abstract

The dinuclear title mol­ecule, [Au_2_Fe(C_10_H_11_N_2_O_3_S)_2_(C_17_H_14_P)_2_]·2CHCl_3_, has crystallographic twofold symmetry with the Fe atom (bonded to two η^5^-cyclo­penta­dienyl rings) situated on the rotation axis. The Au atom exists within a linear geometry defined by an *S*,*P*-donor set with a deviation from linearity [S—Au—P = 176.86 (6)°] due to the close approach of the thio­carbamate O atom [Au⋯O = 3.108 (5) Å]. The mol­ecule has a U-shaped geometry which facilitates the formation of an intra­molecular Au⋯Au inter­action [3.0231 (5) Å]. In the crystal, the presence of C—H⋯O_nitro_ contacts leads to the formation of layers with substantial voids; these are occupied by the solvent mol­ecules of crystallization, which are held in place by C—H⋯S contacts.

## Related literature

For structural systematics and luminescence properties of phosphinegold(I) carbonimidothio­ates, see: Ho *et al.* (2006 ▶); Ho & Tiekink (2007 ▶); Kuan *et al.* (2008 ▶). For the synthesis, see: Hall *et al.* (1993 ▶). 
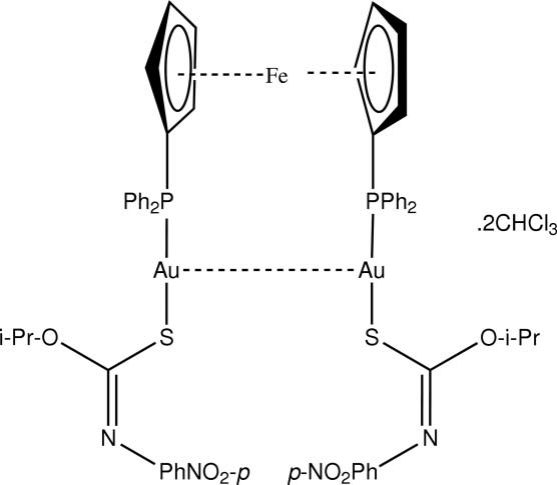


## Experimental

### 

#### Crystal data

[Au_2_Fe(C_10_H_11_N_2_O_3_S)_2_(C_17_H_14_P)_2_]·2CHCl_3_
*M* *_r_* = 1665.56
Monoclinic, 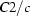

*a* = 25.9661 (13) Å
*b* = 11.5544 (6) Å
*c* = 23.3615 (13) Å
β = 117.293 (1)°
*V* = 6228.7 (6) Å^3^
*Z* = 4
Mo *K*α radiation
μ = 5.36 mm^−1^
*T* = 223 K
0.36 × 0.07 × 0.04 mm

#### Data collection

Bruker SMART CCD diffractometer
Absorption correction: multi-scan (*SADABS*; Bruker, 2000 ▶) *T*_min_ = 0.572, *T*_max_ = 1
21839 measured reflections
7148 independent reflections
4988 reflections with *I* > 2σ(*I*)
*R*_int_ = 0.056

#### Refinement

*R*[*F* ^2^ > 2σ(*F* ^2^)] = 0.045
*wR*(*F* ^2^) = 0.104
*S* = 0.97
7148 reflections
357 parameters
H-atom parameters constrained
Δρ_max_ = 2.08 e Å^−3^
Δρ_min_ = −0.74 e Å^−3^

### 

Data collection: *SMART* (Bruker, 2000 ▶); cell refinement: *SAINT* (Bruker, 2000 ▶); data reduction: *SAINT*; program(s) used to solve structure: *PATTY* in *DIRDIF92* (Beurskens *et al.*, 1992 ▶); program(s) used to refine structure: *SHELXL97* (Sheldrick, 2008 ▶); molecular graphics: *DIAMOND* (Brandenburg, 2006 ▶); software used to prepare material for publication: *SHELXL97*.

## Supplementary Material

Crystal structure: contains datablocks global, I. DOI: 10.1107/S1600536809043864/hb5168sup1.cif

Structure factors: contains datablocks I. DOI: 10.1107/S1600536809043864/hb5168Isup2.hkl

Additional supplementary materials:  crystallographic information; 3D view; checkCIF report

## Figures and Tables

**Table 1:** Selected geometric parameters (Å, °)

Au—S1	2.3127 (16)
Au—P1	2.2594 (15)

**Table 2:** Hydrogen-bond geometry (Å, °)

*D*—H⋯*A*	*D*—H	H⋯*A*	*D*⋯*A*	*D*—H⋯*A*
C21—H21⋯O2^i^	0.94	2.54	3.403 (9)	154
C25—H25⋯O3^ii^	0.94	2.46	3.254 (12)	142
C28—H28⋯S1^iii^	0.99	2.61	3.527 (8)	154

Symmetry codes: (i) 
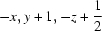
; (ii) 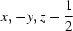; (iii) 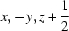.

**Fig. 1.**
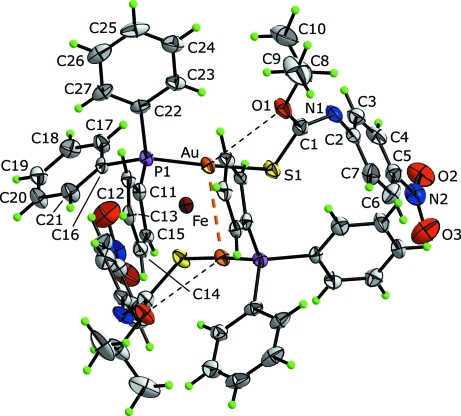
Molecular structure of the dinuclear complex in (I) showing displacement ellipsoids at the 35% probability level. The molecule has twofold symmetry and unlabelled atoms are generated by the symmetry operation -x, y, 1/2 - z.

**Fig. 2.**
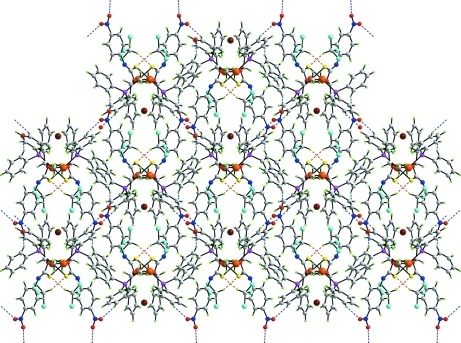
Supramolecular layer formation in (I) mediated by C—H···O contacts (blue dashed lines). The chloroform molecules are connected via C—H···S contacts (orange dashed lines). Colour code: Au, orange; Cl, cyan; S, yellow; P, pink; O, red; N, blue; C, grey; and H, green.

## supplementary crystallographic information

### Comment

The dinuclear title compound, dppf{Au[SC(O-^i^Pr)NC_6_H_4_NO_2_-p]}_2_, was investigated as a part of systematic studies of phosphinegold(I) thiocarbamides (Ho *et al.* 2006; Ho & Tiekink, 2007; Kuan *et al.*, 2008). Crystals were isolated as the di-chloroform solvate, (I), and are isomorphous with the methoxy analogue (Ho *et al.*, 2006). The dinuclear molecule has crystallographic twofold symmetry with the Fe atom lying on the axis; the asymmetric unit comprises half a dinuclear molecule and one chloroform molecule. The gold atom exists in the expected linear geometry defined by a SP donor set, Table 1, and the deviation from linearity is ascribed to the close approach of the O1 atom, Au···O = 3.108 (5) Å. The anion, with a *Z* configuration about the C1═N1 bond, shows the expected characteristics. Overall, the molecule has a U-shaped configuration which allows for the formation of an aurophilic interaction, Au···Au^i^ = 3.0231 (5) Å; (i): -*x*, *y*, 1/2 - *z*.

In the crystal structure of (I), supramolecular layers are formed in the *bc* plane that are mediated by C—H···O interactions, Table 2 and Fig. 2. The resulting framework has solvent accessible voids and these are occupied by the chloroform molecules which are connected *via* C—H···S contacts, Table 2. Layer thus formed stack along the a direction.

### Experimental

The unsolvated compound was prepared following the standard literature procedure from the reaction of dppf(AuCl)_2_ and i-PrOC(═S)N(H)C_6_H_4_NO_2_-4 in the presence of base (Hall *et al.*, 1993); m. pt. 409–410 K. Analysis for C_54_H_50_Au_2_FeN_4_O_6_P_2_S_2_: found (calculated): C: 45.64 (45.45); H: 3.49 (3.53). IR (cm^-1^): ν(C—S) 1100 s, 899*m*; ν(C—N) 1585 s; ν(C—O) 1153 s. ^31^P{^1^H} NMR: δ 32.0 p.p.m. Orange crystals of the di-chloroform solvate (I) were obtained from the layering of ethanol on a chloroform solution of the characterized product.

### Refinement

The H atoms were geometrically placed (C—H = 0.94–0.99 Å) and refined as riding with *U*_iso_(H) = 1.2–1.5*U*_eq_(C). The maximum and minimum residual electron density peaks of 2.08 and 0.74 e Å^-3^, respectively, were located 0.83 Å and 0.74 Å from the Au atom.

### Crystal data

**Table:**

[Au_2_Fe(C_10_H_11_N_2_O_3_S)_2_(C_17_H_14_P)_2_]·2CHCl_3_	*F*(000) = 3248
*M**_r_* = 1665.56	*D*_x_ = 1.776 Mg m^−^^3^
Monoclinic, *C*2/*c*	Mo *K*α radiation, λ = 0.71069 Å
Hall symbol: -C 2yc	Cell parameters from 3297 reflections
*a* = 25.9661 (13) Å	θ = 4.4–23.2°
*b* = 11.5544 (6) Å	µ = 5.36 mm^−^^1^
*c* = 23.3615 (13) Å	*T* = 223 K
β = 117.293 (1)°	Needle, orange
*V* = 6228.7 (6) Å^3^	0.36 × 0.07 × 0.04 mm
*Z* = 4	

### Data collection

**Table:**

Bruker SMART CCD diffractometer	7148 independent reflections
Radiation source: fine-focus sealed tube	4988 reflections with *I* > 2σ(*I*)
graphite	*R*_int_ = 0.056
ω scans	θ_max_ = 27.5°, θ_min_ = 1.8°
Absorption correction: multi-scan (*SADABS*; Bruker, 2000)	*h* = −31→33
*T*_min_ = 0.572, *T*_max_ = 1	*k* = −15→15
21839 measured reflections	*l* = −30→16

### Refinement

**Table:**

Refinement on *F*^2^	Primary atom site location: structure-invariant direct methods
Least-squares matrix: full	Secondary atom site location: difference Fourier map
*R*[*F*^2^ > 2σ(*F*^2^)] = 0.045	Hydrogen site location: inferred from neighbouring sites
*wR*(*F*^2^) = 0.104	H-atom parameters constrained
*S* = 0.97	*w* = 1/[σ^2^(*F*_o_^2^) + (0.0493*P*)^2^] where *P* = (*F*_o_^2^ + 2*F*_c_^2^)/3
7148 reflections	(Δ/σ)_max_ = 0.001
357 parameters	Δρ_max_ = 2.08 e Å^−^^3^
0 restraints	Δρ_min_ = −0.74 e Å^−^^3^

### Special details

**Table:**

Geometry. All s.u.'s (except the s.u. in the dihedral angle between two l.s. planes) are estimated using the full covariance matrix. The cell s.u.'s are taken into account individually in the estimation of s.u.'s in distances, angles and torsion angles; correlations between s.u.'s in cell parameters are only used when they are defined by crystal symmetry. An approximate (isotropic) treatment of cell s.u.'s is used for estimating s.u.'s involving l.s. planes.
Refinement. Refinement of *F*^2^ against ALL reflections. The weighted *R*-factor *wR* and goodness of fit *S* are based on *F*^2^, conventional *R*-factors *R* are based on *F*, with *F* set to zero for negative *F*^2^. The threshold expression of *F*^2^ > 2σ(*F*^2^) is used only for calculating *R*-factors(gt) *etc*. and is not relevant to the choice of reflections for refinement. *R*-factors based on *F*^2^ are statistically about twice as large as those based on *F*, and *R*- factors based on ALL data will be even larger.

### Fractional atomic coordinates and isotropic or equivalent isotropic displacement parameters (Å^2^)

**Table:**

	*x*	*y*	*z*	*U*_iso_*/*U*_eq_	
Au	0.038384 (9)	0.040380 (18)	0.217132 (11)	0.03822 (9)	
Fe	0.0000	0.36727 (10)	0.2500	0.0348 (3)	
Cl1	0.09462 (11)	0.1755 (2)	0.62226 (16)	0.1115 (10)	
Cl2	0.09451 (11)	0.4042 (2)	0.66861 (19)	0.1263 (13)	
Cl3	−0.00491 (9)	0.2607 (2)	0.63203 (13)	0.0870 (7)	
S1	0.08961 (7)	−0.09108 (14)	0.29813 (8)	0.0475 (4)	
P1	−0.00697 (6)	0.17293 (13)	0.13880 (7)	0.0354 (3)	
O1	0.17098 (19)	0.0531 (4)	0.3072 (2)	0.0568 (13)	
O2	0.1974 (2)	−0.6234 (5)	0.4520 (3)	0.0835 (19)	
O3	0.1523 (4)	−0.5385 (5)	0.4984 (3)	0.097 (2)	
N1	0.2054 (2)	−0.1139 (5)	0.3638 (3)	0.0548 (15)	
N2	0.1784 (3)	−0.5363 (5)	0.4654 (3)	0.0637 (18)	
C1	0.1632 (3)	−0.0518 (5)	0.3277 (3)	0.0458 (16)	
C2	0.1963 (2)	−0.2195 (6)	0.3880 (3)	0.0480 (17)	
C3	0.2107 (3)	−0.3236 (6)	0.3689 (4)	0.0560 (18)	
H3	0.2246	−0.3233	0.3381	0.067*	
C4	0.2049 (3)	−0.4253 (6)	0.3942 (4)	0.0542 (19)	
H4	0.2147	−0.4951	0.3810	0.065*	
C5	0.1849 (3)	−0.4263 (6)	0.4384 (3)	0.0509 (17)	
C6	0.1698 (3)	−0.3254 (6)	0.4587 (3)	0.0588 (19)	
H6	0.1557	−0.3275	0.4893	0.071*	
C7	0.1757 (3)	−0.2224 (6)	0.4334 (3)	0.0556 (19)	
H7	0.1658	−0.1530	0.4468	0.067*	
C8	0.2304 (3)	0.0878 (8)	0.3263 (5)	0.075 (3)	
H8	0.2569	0.0509	0.3678	0.089*	
C9	0.2330 (4)	0.2145 (8)	0.3331 (6)	0.113 (4)	
H9A	0.2254	0.2364	0.3686	0.170*	
H9B	0.2042	0.2493	0.2936	0.170*	
H9C	0.2713	0.2414	0.3415	0.170*	
C10	0.2436 (4)	0.0462 (9)	0.2737 (6)	0.107 (4)	
H10A	0.2424	−0.0377	0.2723	0.160*	
H10B	0.2819	0.0724	0.2820	0.160*	
H10C	0.2150	0.0769	0.2327	0.160*	
C11	−0.0392 (2)	0.2915 (5)	0.1610 (3)	0.0340 (13)	
C12	−0.0298 (2)	0.4126 (5)	0.1557 (3)	0.0378 (14)	
H12	−0.0075	0.4446	0.1373	0.045*	
C13	−0.0598 (3)	0.4751 (5)	0.1828 (3)	0.0445 (16)	
H13	−0.0610	0.5561	0.1855	0.053*	
C14	−0.0877 (3)	0.3958 (5)	0.2052 (3)	0.0445 (15)	
H14	−0.1104	0.4149	0.2256	0.053*	
C15	−0.0756 (2)	0.2828 (5)	0.1919 (3)	0.0354 (13)	
H15	−0.0891	0.2137	0.2016	0.042*	
C16	−0.0634 (2)	0.1105 (5)	0.0653 (3)	0.0342 (13)	
C17	−0.0487 (3)	0.0138 (5)	0.0404 (3)	0.0483 (17)	
H17	−0.0105	−0.0142	0.0606	0.058*	
C18	−0.0892 (3)	−0.0405 (6)	−0.0132 (4)	0.0547 (18)	
H18	−0.0791	−0.1059	−0.0298	0.066*	
C19	−0.1452 (3)	0.0012 (6)	−0.0428 (3)	0.0529 (18)	
H19	−0.1732	−0.0353	−0.0800	0.063*	
C20	−0.1603 (3)	0.0951 (6)	−0.0183 (3)	0.0569 (19)	
H20	−0.1987	0.1221	−0.0383	0.068*	
C21	−0.1191 (3)	0.1504 (5)	0.0357 (3)	0.0499 (17)	
H21	−0.1294	0.2156	0.0522	0.060*	
C22	0.0426 (3)	0.2447 (5)	0.1154 (3)	0.0423 (15)	
C23	0.1015 (3)	0.2386 (6)	0.1572 (3)	0.0490 (16)	
H23	0.1150	0.1944	0.1951	0.059*	
C24	0.1405 (3)	0.2984 (7)	0.1426 (4)	0.068 (2)	
H24	0.1804	0.2935	0.1705	0.081*	
C25	0.1213 (4)	0.3646 (6)	0.0878 (4)	0.070 (2)	
H25	0.1477	0.4071	0.0790	0.084*	
C26	0.0623 (4)	0.3680 (6)	0.0455 (4)	0.064 (2)	
H26	0.0489	0.4108	0.0071	0.077*	
C27	0.0234 (3)	0.3085 (5)	0.0599 (3)	0.0525 (17)	
H27	−0.0165	0.3119	0.0314	0.063*	
C28	0.0705 (3)	0.2630 (6)	0.6651 (4)	0.062 (2)	
H28	0.0867	0.2328	0.7096	0.075*	

### Atomic displacement parameters (Å^2^)

**Table:**

	*U*^11^	*U*^22^	*U*^33^	*U*^12^	*U*^13^	*U*^23^
Au	0.03377 (13)	0.03382 (12)	0.04130 (14)	0.00501 (10)	0.01225 (10)	0.00307 (11)
Fe	0.0327 (6)	0.0341 (6)	0.0337 (6)	0.000	0.0118 (5)	0.000
Cl1	0.0976 (18)	0.1016 (18)	0.161 (3)	−0.0139 (15)	0.0819 (19)	−0.0543 (19)
Cl2	0.0878 (17)	0.0519 (12)	0.239 (4)	−0.0138 (12)	0.074 (2)	−0.0203 (18)
Cl3	0.0653 (13)	0.0782 (14)	0.121 (2)	−0.0069 (11)	0.0461 (14)	−0.0200 (14)
S1	0.0364 (8)	0.0424 (8)	0.0557 (10)	0.0049 (7)	0.0142 (8)	0.0126 (8)
P1	0.0335 (8)	0.0328 (7)	0.0369 (8)	0.0014 (6)	0.0135 (7)	−0.0007 (6)
O1	0.037 (2)	0.048 (3)	0.072 (3)	−0.003 (2)	0.014 (2)	0.017 (2)
O2	0.073 (4)	0.041 (3)	0.136 (6)	−0.006 (3)	0.047 (4)	0.004 (3)
O3	0.152 (7)	0.061 (4)	0.103 (5)	−0.014 (4)	0.079 (5)	−0.004 (3)
N1	0.030 (3)	0.058 (3)	0.068 (4)	0.007 (3)	0.015 (3)	0.019 (3)
N2	0.060 (4)	0.046 (4)	0.075 (5)	−0.010 (3)	0.022 (4)	0.001 (3)
C1	0.044 (4)	0.045 (4)	0.046 (4)	0.000 (3)	0.019 (3)	0.005 (3)
C2	0.029 (3)	0.055 (4)	0.051 (4)	0.005 (3)	0.011 (3)	0.008 (3)
C3	0.044 (4)	0.061 (4)	0.066 (5)	0.008 (3)	0.028 (4)	0.006 (4)
C4	0.039 (4)	0.046 (4)	0.076 (5)	0.005 (3)	0.024 (4)	−0.001 (4)
C5	0.043 (4)	0.050 (4)	0.053 (4)	−0.003 (3)	0.015 (3)	0.003 (3)
C6	0.077 (5)	0.052 (4)	0.051 (4)	−0.006 (4)	0.032 (4)	0.002 (3)
C7	0.064 (5)	0.042 (4)	0.055 (4)	0.011 (3)	0.023 (4)	0.002 (3)
C8	0.035 (4)	0.079 (6)	0.091 (6)	0.000 (4)	0.013 (4)	0.030 (5)
C9	0.065 (6)	0.091 (7)	0.146 (10)	−0.037 (5)	0.016 (6)	−0.003 (7)
C10	0.066 (6)	0.108 (8)	0.159 (11)	0.005 (5)	0.063 (7)	0.021 (8)
C11	0.035 (3)	0.031 (3)	0.028 (3)	0.003 (2)	0.008 (3)	0.001 (2)
C12	0.038 (3)	0.034 (3)	0.035 (3)	−0.001 (2)	0.011 (3)	0.003 (3)
C13	0.044 (3)	0.037 (3)	0.041 (3)	0.007 (3)	0.010 (3)	0.003 (3)
C14	0.035 (3)	0.051 (4)	0.042 (4)	0.007 (3)	0.013 (3)	−0.002 (3)
C15	0.023 (3)	0.043 (3)	0.035 (3)	−0.002 (2)	0.010 (2)	0.003 (3)
C16	0.035 (3)	0.032 (3)	0.030 (3)	0.002 (2)	0.011 (2)	0.005 (2)
C17	0.043 (4)	0.046 (4)	0.050 (4)	0.009 (3)	0.017 (3)	−0.004 (3)
C18	0.060 (4)	0.045 (4)	0.062 (4)	−0.001 (3)	0.031 (4)	−0.013 (3)
C19	0.043 (4)	0.061 (4)	0.047 (4)	−0.014 (3)	0.015 (3)	−0.015 (3)
C20	0.036 (3)	0.062 (4)	0.056 (4)	0.005 (3)	0.006 (3)	−0.009 (4)
C21	0.048 (4)	0.039 (3)	0.057 (4)	0.007 (3)	0.019 (3)	−0.008 (3)
C22	0.052 (4)	0.034 (3)	0.051 (4)	0.000 (3)	0.033 (3)	−0.006 (3)
C23	0.044 (4)	0.048 (4)	0.057 (4)	−0.007 (3)	0.026 (3)	−0.009 (3)
C24	0.055 (4)	0.082 (6)	0.075 (6)	−0.013 (4)	0.037 (4)	−0.016 (5)
C25	0.097 (6)	0.052 (4)	0.097 (7)	−0.032 (4)	0.076 (6)	−0.029 (5)
C26	0.103 (7)	0.046 (4)	0.068 (5)	−0.005 (4)	0.060 (5)	−0.003 (4)
C27	0.076 (5)	0.042 (4)	0.053 (4)	−0.001 (3)	0.041 (4)	−0.006 (3)
C28	0.054 (4)	0.047 (4)	0.084 (6)	0.005 (3)	0.030 (4)	0.000 (4)

### Geometric parameters (Å, °)

**Table:**

Au—S1	2.3127 (16)	C8—H8	0.9900
Au—P1	2.2594 (15)	C9—H9A	0.9700
Au—Au^i^	3.0231 (5)	C9—H9B	0.9700
Fe—C12^i^	2.041 (6)	C9—H9C	0.9700
Fe—C12	2.041 (6)	C10—H10A	0.9700
Fe—C11	2.045 (5)	C10—H10B	0.9700
Fe—C11^i^	2.045 (5)	C10—H10C	0.9700
Fe—C13^i^	2.047 (6)	C11—C15	1.431 (8)
Fe—C13	2.047 (6)	C11—C12	1.436 (8)
Fe—C14^i^	2.050 (6)	C12—C13	1.407 (9)
Fe—C14	2.050 (6)	C12—H12	0.9400
Fe—C15	2.051 (5)	C13—C14	1.409 (9)
Fe—C15^i^	2.051 (5)	C13—H13	0.9400
Cl1—C28	1.729 (8)	C14—C15	1.411 (8)
Cl2—C28	1.736 (7)	C14—H14	0.9400
Cl3—C28	1.744 (7)	C15—H15	0.9400
S1—C1	1.767 (6)	C16—C21	1.365 (8)
P1—C11	1.802 (6)	C16—C17	1.392 (8)
P1—C22	1.812 (6)	C17—C18	1.363 (9)
P1—C16	1.818 (5)	C17—H17	0.9400
O1—C1	1.353 (7)	C18—C19	1.380 (9)
O1—C8	1.453 (8)	C18—H18	0.9400
O2—N2	1.223 (8)	C19—C20	1.363 (10)
O3—N2	1.239 (9)	C19—H19	0.9400
N1—C1	1.257 (7)	C20—C21	1.382 (8)
N1—C2	1.410 (8)	C20—H20	0.9400
N2—C5	1.463 (9)	C21—H21	0.9400
C2—C7	1.390 (10)	C22—C27	1.372 (9)
C2—C3	1.392 (9)	C22—C23	1.389 (8)
C3—C4	1.355 (9)	C23—C24	1.390 (10)
C3—H3	0.9400	C23—H23	0.9400
C4—C5	1.353 (10)	C24—C25	1.373 (11)
C4—H4	0.9400	C24—H24	0.9400
C5—C6	1.381 (10)	C25—C26	1.391 (11)
C6—C7	1.369 (9)	C25—H25	0.9400
C6—H6	0.9400	C26—C27	1.386 (9)
C7—H7	0.9400	C26—H26	0.9400
C8—C9	1.470 (12)	C27—H27	0.9400
C8—C10	1.498 (14)	C28—H28	0.9900
			
P1—Au—S1	176.86 (6)	C10—C8—H8	110.3
P1—Au—Au^i^	101.03 (4)	C8—C9—H9A	109.5
S1—Au—Au^i^	81.38 (5)	C8—C9—H9B	109.5
C12^i^—Fe—C12	150.2 (3)	H9A—C9—H9B	109.5
C12^i^—Fe—C11	166.6 (2)	C8—C9—H9C	109.5
C12—Fe—C11	41.1 (2)	H9A—C9—H9C	109.5
C12^i^—Fe—C11^i^	41.1 (2)	H9B—C9—H9C	109.5
C12—Fe—C11^i^	166.6 (2)	C8—C10—H10A	109.5
C11—Fe—C11^i^	129.3 (3)	C8—C10—H10B	109.5
C12^i^—Fe—C13^i^	40.3 (3)	H10A—C10—H10B	109.5
C12—Fe—C13^i^	116.8 (2)	C8—C10—H10C	109.5
C11—Fe—C13^i^	152.7 (3)	H10A—C10—H10C	109.5
C11^i^—Fe—C13^i^	68.4 (2)	H10B—C10—H10C	109.5
C12^i^—Fe—C13	116.8 (2)	C15—C11—C12	106.9 (5)
C12—Fe—C13	40.3 (2)	C15—C11—P1	126.4 (4)
C11—Fe—C13	68.4 (2)	C12—C11—P1	126.6 (5)
C11^i^—Fe—C13	152.7 (3)	C15—C11—Fe	69.8 (3)
C13^i^—Fe—C13	105.0 (3)	C12—C11—Fe	69.3 (3)
C12^i^—Fe—C14^i^	68.0 (3)	P1—C11—Fe	122.5 (3)
C12—Fe—C14^i^	107.0 (3)	C13—C12—C11	108.0 (6)
C11—Fe—C14^i^	120.4 (2)	C13—C12—Fe	70.1 (4)
C11^i^—Fe—C14^i^	68.4 (2)	C11—C12—Fe	69.6 (3)
C13^i^—Fe—C14^i^	40.2 (3)	C13—C12—H12	126.0
C13—Fe—C14^i^	124.6 (3)	C11—C12—H12	126.0
C12^i^—Fe—C14	107.0 (3)	Fe—C12—H12	125.9
C12—Fe—C14	68.0 (3)	C12—C13—C14	108.6 (5)
C11—Fe—C14	68.4 (2)	C12—C13—Fe	69.6 (3)
C11^i^—Fe—C14	120.4 (2)	C14—C13—Fe	70.0 (3)
C13^i^—Fe—C14	124.6 (3)	C12—C13—H13	125.7
C13—Fe—C14	40.2 (3)	C14—C13—H13	125.7
C14^i^—Fe—C14	161.5 (4)	Fe—C13—H13	126.3
C12^i^—Fe—C15	127.7 (2)	C13—C14—C15	108.4 (6)
C12—Fe—C15	68.5 (2)	C13—C14—Fe	69.8 (3)
C11—Fe—C15	40.9 (2)	C15—C14—Fe	69.9 (3)
C11^i^—Fe—C15	110.4 (2)	C13—C14—H14	125.8
C13^i^—Fe—C15	163.0 (3)	C15—C14—H14	125.8
C13—Fe—C15	67.8 (2)	Fe—C14—H14	126.1
C14^i^—Fe—C15	156.4 (3)	C14—C15—C11	108.1 (5)
C14—Fe—C15	40.2 (2)	C14—C15—Fe	69.8 (3)
C12^i^—Fe—C15^i^	68.5 (2)	C11—C15—Fe	69.4 (3)
C12—Fe—C15^i^	127.7 (2)	C14—C15—H15	125.9
C11—Fe—C15^i^	110.4 (2)	C11—C15—H15	125.9
C11^i^—Fe—C15^i^	40.9 (2)	Fe—C15—H15	126.4
C13^i^—Fe—C15^i^	67.8 (2)	C21—C16—C17	119.2 (5)
C13—Fe—C15^i^	163.0 (3)	C21—C16—P1	123.5 (5)
C14^i^—Fe—C15^i^	40.2 (2)	C17—C16—P1	117.2 (4)
C14—Fe—C15^i^	156.4 (3)	C18—C17—C16	120.6 (6)
C15—Fe—C15^i^	123.1 (3)	C18—C17—H17	119.7
C1—S1—Au	105.3 (2)	C16—C17—H17	119.7
C11—P1—C22	103.1 (3)	C17—C18—C19	119.4 (6)
C11—P1—C16	107.1 (3)	C17—C18—H18	120.3
C22—P1—C16	105.8 (3)	C19—C18—H18	120.3
C11—P1—Au	114.88 (19)	C20—C19—C18	120.4 (6)
C22—P1—Au	112.1 (2)	C20—C19—H19	119.8
C16—P1—Au	113.01 (18)	C18—C19—H19	119.8
C1—O1—C8	116.6 (5)	C19—C20—C21	120.0 (6)
C1—N1—C2	120.5 (5)	C19—C20—H20	120.0
O2—N2—O3	122.9 (7)	C21—C20—H20	120.0
O2—N2—C5	117.7 (8)	C16—C21—C20	120.2 (6)
O3—N2—C5	119.3 (7)	C16—C21—H21	119.9
N1—C1—O1	121.5 (6)	C20—C21—H21	119.9
N1—C1—S1	124.8 (5)	C27—C22—C23	119.8 (6)
O1—C1—S1	113.7 (4)	C27—C22—P1	122.0 (5)
C7—C2—C3	118.6 (6)	C23—C22—P1	118.1 (5)
C7—C2—N1	121.4 (6)	C22—C23—C24	119.6 (7)
C3—C2—N1	120.0 (7)	C22—C23—H23	120.2
C4—C3—C2	120.5 (7)	C24—C23—H23	120.2
C4—C3—H3	119.7	C25—C24—C23	120.7 (7)
C2—C3—H3	119.7	C25—C24—H24	119.6
C5—C4—C3	120.0 (7)	C23—C24—H24	119.6
C5—C4—H4	120.0	C24—C25—C26	119.3 (7)
C3—C4—H4	120.0	C24—C25—H25	120.3
C4—C5—C6	121.6 (7)	C26—C25—H25	120.3
C4—C5—N2	119.8 (7)	C27—C26—C25	120.0 (7)
C6—C5—N2	118.6 (7)	C27—C26—H26	120.0
C7—C6—C5	118.7 (7)	C25—C26—H26	120.0
C7—C6—H6	120.7	C22—C27—C26	120.5 (7)
C5—C6—H6	120.7	C22—C27—H27	119.8
C6—C7—C2	120.6 (7)	C26—C27—H27	119.8
C6—C7—H7	119.7	Cl1—C28—Cl2	111.1 (5)
C2—C7—H7	119.7	Cl1—C28—Cl3	111.5 (4)
O1—C8—C9	107.3 (7)	Cl2—C28—Cl3	109.9 (4)
O1—C8—C10	105.6 (7)	Cl1—C28—H28	108.1
C9—C8—C10	113.0 (9)	Cl2—C28—H28	108.1
O1—C8—H8	110.3	Cl3—C28—H28	108.1
C9—C8—H8	110.3		
			
Au^i^—Au—S1—C1	−136.1 (2)	C11—Fe—C13—C12	−38.2 (3)
Au^i^—Au—P1—C11	35.1 (2)	C11^i^—Fe—C13—C12	−174.3 (4)
Au^i^—Au—P1—C22	152.3 (2)	C13^i^—Fe—C13—C12	113.9 (4)
Au^i^—Au—P1—C16	−88.2 (2)	C14^i^—Fe—C13—C12	74.6 (4)
C2—N1—C1—O1	175.8 (6)	C14—Fe—C13—C12	−119.8 (5)
C2—N1—C1—S1	−5.1 (10)	C15—Fe—C13—C12	−82.4 (4)
C8—O1—C1—N1	2.9 (10)	C15^i^—Fe—C13—C12	51.0 (9)
C8—O1—C1—S1	−176.2 (6)	C12^i^—Fe—C13—C14	−84.9 (4)
Au—S1—C1—N1	−166.5 (6)	C12—Fe—C13—C14	119.8 (5)
Au—S1—C1—O1	12.6 (5)	C11—Fe—C13—C14	81.6 (4)
C1—N1—C2—C7	−69.1 (9)	C11^i^—Fe—C13—C14	−54.4 (6)
C1—N1—C2—C3	114.0 (8)	C13^i^—Fe—C13—C14	−126.3 (4)
C7—C2—C3—C4	−0.2 (10)	C14^i^—Fe—C13—C14	−165.5 (3)
N1—C2—C3—C4	176.8 (6)	C15—Fe—C13—C14	37.4 (4)
C2—C3—C4—C5	0.0 (10)	C15^i^—Fe—C13—C14	170.9 (7)
C3—C4—C5—C6	0.3 (11)	C12—C13—C14—C15	−0.4 (7)
C3—C4—C5—N2	179.7 (6)	Fe—C13—C14—C15	−59.5 (4)
O2—N2—C5—C4	7.8 (10)	C12—C13—C14—Fe	59.1 (4)
O3—N2—C5—C4	−169.4 (7)	C12^i^—Fe—C14—C13	111.6 (4)
O2—N2—C5—C6	−172.8 (7)	C12—Fe—C14—C13	−37.2 (4)
O3—N2—C5—C6	10.0 (10)	C11—Fe—C14—C13	−81.7 (4)
C4—C5—C6—C7	−0.4 (11)	C11^i^—Fe—C14—C13	154.4 (4)
N2—C5—C6—C7	−179.8 (6)	C13^i^—Fe—C14—C13	70.9 (6)
C5—C6—C7—C2	0.2 (11)	C14^i^—Fe—C14—C13	40.4 (3)
C3—C2—C7—C6	0.1 (10)	C15—Fe—C14—C13	−119.5 (5)
N1—C2—C7—C6	−176.8 (6)	C15^i^—Fe—C14—C13	−173.3 (5)
C1—O1—C8—C9	−148.5 (8)	C12^i^—Fe—C14—C15	−128.9 (4)
C1—O1—C8—C10	90.7 (8)	C12—Fe—C14—C15	82.3 (4)
C22—P1—C11—C15	−170.5 (5)	C11—Fe—C14—C15	37.8 (4)
C16—P1—C11—C15	78.1 (5)	C11^i^—Fe—C14—C15	−86.1 (4)
Au—P1—C11—C15	−48.3 (5)	C13^i^—Fe—C14—C15	−169.6 (4)
C22—P1—C11—C12	4.3 (6)	C13—Fe—C14—C15	119.5 (5)
C16—P1—C11—C12	−107.1 (5)	C14^i^—Fe—C14—C15	159.9 (4)
Au—P1—C11—C12	126.6 (4)	C15^i^—Fe—C14—C15	−53.8 (9)
C22—P1—C11—Fe	−82.8 (4)	C13—C14—C15—C11	0.4 (6)
C16—P1—C11—Fe	165.8 (3)	Fe—C14—C15—C11	−59.0 (4)
Au—P1—C11—Fe	39.4 (4)	C13—C14—C15—Fe	59.4 (4)
C12^i^—Fe—C11—C15	34.7 (11)	C12—C11—C15—C14	−0.3 (6)
C12—Fe—C11—C15	−118.1 (5)	P1—C11—C15—C14	175.4 (4)
C11^i^—Fe—C11—C15	75.1 (3)	Fe—C11—C15—C14	59.3 (4)
C13^i^—Fe—C11—C15	−161.5 (5)	C12—C11—C15—Fe	−59.6 (4)
C13—Fe—C11—C15	−80.7 (4)	P1—C11—C15—Fe	116.1 (4)
C14^i^—Fe—C11—C15	160.9 (3)	C12^i^—Fe—C15—C14	70.1 (5)
C14—Fe—C11—C15	−37.2 (3)	C12—Fe—C15—C14	−80.9 (4)
C15^i^—Fe—C11—C15	117.5 (4)	C11—Fe—C15—C14	−119.5 (5)
C12^i^—Fe—C11—C12	152.8 (8)	C11^i^—Fe—C15—C14	113.4 (4)
C11^i^—Fe—C11—C12	−166.8 (4)	C13^i^—Fe—C15—C14	30.7 (10)
C13^i^—Fe—C11—C12	−43.4 (6)	C13—Fe—C15—C14	−37.4 (4)
C13—Fe—C11—C12	37.4 (4)	C14^i^—Fe—C15—C14	−164.2 (4)
C14^i^—Fe—C11—C12	−81.0 (4)	C15^i^—Fe—C15—C14	157.3 (4)
C14—Fe—C11—C12	80.9 (4)	C12^i^—Fe—C15—C11	−170.4 (3)
C15—Fe—C11—C12	118.1 (5)	C12—Fe—C15—C11	38.6 (3)
C15^i^—Fe—C11—C12	−124.4 (4)	C11^i^—Fe—C15—C11	−127.1 (4)
C12^i^—Fe—C11—P1	−86.2 (11)	C13^i^—Fe—C15—C11	150.2 (8)
C12—Fe—C11—P1	120.9 (5)	C13—Fe—C15—C11	82.1 (4)
C11^i^—Fe—C11—P1	−45.9 (3)	C14^i^—Fe—C15—C11	−44.7 (7)
C13^i^—Fe—C11—P1	77.5 (6)	C14—Fe—C15—C11	119.5 (5)
C13—Fe—C11—P1	158.4 (4)	C15^i^—Fe—C15—C11	−83.2 (3)
C14^i^—Fe—C11—P1	40.0 (5)	C11—P1—C16—C21	−0.6 (6)
C14—Fe—C11—P1	−158.2 (4)	C22—P1—C16—C21	−110.1 (6)
C15—Fe—C11—P1	−121.0 (5)	Au—P1—C16—C21	126.9 (5)
C15^i^—Fe—C11—P1	−3.5 (4)	C11—P1—C16—C17	−177.7 (5)
C15—C11—C12—C13	0.1 (6)	C22—P1—C16—C17	72.8 (5)
P1—C11—C12—C13	−175.6 (4)	Au—P1—C16—C17	−50.2 (5)
Fe—C11—C12—C13	−59.8 (4)	C21—C16—C17—C18	0.2 (10)
C15—C11—C12—Fe	59.9 (4)	P1—C16—C17—C18	177.4 (6)
P1—C11—C12—Fe	−115.8 (4)	C16—C17—C18—C19	0.2 (11)
C12^i^—Fe—C12—C13	−48.7 (3)	C17—C18—C19—C20	−0.9 (12)
C11—Fe—C12—C13	119.0 (5)	C18—C19—C20—C21	1.2 (12)
C11^i^—Fe—C12—C13	168.6 (9)	C17—C16—C21—C20	0.1 (10)
C13^i^—Fe—C12—C13	−81.6 (5)	P1—C16—C21—C20	−176.9 (6)
C14^i^—Fe—C12—C13	−123.9 (4)	C19—C20—C21—C16	−0.8 (11)
C14—Fe—C12—C13	37.2 (4)	C11—P1—C22—C27	−70.8 (6)
C15—Fe—C12—C13	80.7 (4)	C16—P1—C22—C27	41.6 (6)
C15^i^—Fe—C12—C13	−163.3 (4)	Au—P1—C22—C27	165.1 (5)
C12^i^—Fe—C12—C11	−167.7 (4)	C11—P1—C22—C23	106.0 (5)
C11^i^—Fe—C12—C11	49.5 (13)	C16—P1—C22—C23	−141.7 (5)
C13^i^—Fe—C12—C11	159.4 (3)	Au—P1—C22—C23	−18.1 (5)
C13—Fe—C12—C11	−119.0 (5)	C27—C22—C23—C24	0.7 (10)
C14^i^—Fe—C12—C11	117.1 (4)	P1—C22—C23—C24	−176.2 (5)
C14—Fe—C12—C11	−81.8 (4)	C22—C23—C24—C25	0.9 (11)
C15—Fe—C12—C11	−38.4 (3)	C23—C24—C25—C26	−2.4 (11)
C15^i^—Fe—C12—C11	77.6 (4)	C24—C25—C26—C27	2.3 (11)
C11—C12—C13—C14	0.2 (6)	C23—C22—C27—C26	−0.7 (9)
Fe—C12—C13—C14	−59.3 (4)	P1—C22—C27—C26	176.0 (5)
C11—C12—C13—Fe	59.5 (4)	C25—C26—C27—C22	−0.8 (10)
C12^i^—Fe—C13—C12	155.3 (3)		

Symmetry codes: (i) −*x*, *y*, −*z*+1/2.

### Hydrogen-bond geometry (Å, °)

**Table:**

*D*—H···*A*	*D*—H	H···*A*	*D*···*A*	*D*—H···*A*
C21—H21···O2^ii^	0.94	2.54	3.403 (9)	154
C25—H25···O3^iii^	0.94	2.46	3.254 (12)	142
C28—H28···S1^iv^	0.99	2.61	3.527 (8)	154

Symmetry codes: (ii) −*x*, *y*+1, −*z*+1/2; (iii) *x*, −*y*, *z*−1/2; (iv) *x*, −*y*, *z*+1/2.
